# Rare Case of Laurence–Moon–Bardet–Biedl Syndrome With Pulmonary Hypertension: A Case Report

**DOI:** 10.1002/ccr3.70691

**Published:** 2025-07-29

**Authors:** Areeba Mariam Mehmood, S. M. Salman Hassan, Ayesha Malik, Muhammad Faizan, Muhammad Usama bin Shabbir, Aqsa Jabeen, Allahdad Khan, Aseel Kamal

**Affiliations:** ^1^ Sargodha Medical College, University of Sargodha Sargodha Pakistan; ^2^ Department of Medicine Nishtar Medical University Multan Pakistan; ^3^ Pakistan Institute of Medical Sciences Islamabad Pakistan; ^4^ Faculty of Medicine University of Gezira Wad Madani Sudan

**Keywords:** ciliopathy, Laurence–Moon–Bardet–Biedl syndrome, mental retardation, metabolic abnormalities, pulmonary hypertension

## Abstract

Laurence‐Moon‐Bardet‐Biedl Syndrome is a complicated polygenic disorder that can have lethal manifestations such as pulmonary hypertension. Early recognition, comprehensive medical treatment, and psychological intervention for the family are crucial, but survival is usually short in those with a poor functional status and increasingly impaired health.

## 1 Introduction

Laurence–Moon–Bardet–Biedl Syndrome (LMBBS) is a rare disorder with a prevalence of 1/15,000 worldwide [1]. It is a primary ciliopathy associated with mutations in multiple genes, resulting in defective signal transduction pathways and developmental abnormalities [2].

It is composed of a constellation of symptoms with a multi‐system involvement, including renal dysfunction, retinal dystrophy, and skeletal abnormalities; namely polydactyly, cognitive dysfunction, hypogonadism, metabolic abnormalities, including type 2 diabetes, and obesity [3, 4].

Forsythe et al. [2] mentioned that the diagnosis of Bardet–Biedl Syndrome is made after the patient is presented with visual problems that involve dystrophy of rods and cones. The diagnosis can also be made early if the patient has polydactyly and structural kidney anomalies.

Due to the variability present in the dysmorphic presentation of the syndrome, a modified diagnostic criterion was given by Beales et al. [5] to make a clinical diagnosis of the syndrome. According to the criteria, 4 primary features or 3 primary +2 secondary features are required to make the diagnosis. The features are given in Table 1 [4]. A sign of (+) indicates the features present in the patient. Genetic testing can confirm the disease, but it is not required if the clinical criteria for the diagnosis are met [6].

**TABLE 1:** Criteria for LMBBS with positive features in the patient.

Primary characteristics	Secondary characteristics
Rod cone dystrophy (+)	Ataxia/poor coordination (+)
Renal anomalies (+)	Developmental delay (+)
Learning difficulties (+)	Syndactyly/brachydactyly
Genital anomalies	Speech delay
Obesity (+)	Hyposmia/anosmia
Polydactyly	Congenital heart disease
	Diabetes mellitus
	Dental anomalies

LMBBS follows an autosomal recessive pattern of inheritance, with at least 26 associated genes identified as BBS1–BBS22, CEP 19, NPHP1, SCAPER, and SCLT1 [7]. However, an oligogenic mode of inheritance has also been observed [8]. Due to significant phenotypic overlap between ciliopathies, which is likely explained by the underlying allelic makeup, the more recent term, Bardet–Biedl syndrome (BBS), has replaced the older LMBBS. To date, 21 BBS genes (BBS1–BBS20 and NPHP1) have been identified [5], but ongoing research of unstudied populations and the discovery of whole‐exome sequencing may increase this number in the near future. These BBS proteins localize to the centrosome, which regulates the ciliary transport. Disruption of this function classifies BBS as a ciliopathy, explaining its multisystem involvement [5]. This syndrome is most often noticed in children as a result of consanguineous marriages [9, 10].

While most of the patients present with visual problems, renal anomalies, or mental retardation as the main presentation [1], our case is unique as our patient presented with the primary complaint of shortness of breath and increased respiratory rate that later was attributed to pulmonary hypertension, which is a rare presentation of the syndrome. This atypical presentation underscores the need to recognize LMBBS as a systemic disorder with diverse clinical expressions.

This case highlights the importance of considering BBS in patients with unexplained pulmonary hypertension and contributes to the growing body of evidence regarding the syndrome's variable phenotype. Further research is needed to elucidate the underlying mechanisms linking BBS to pulmonary involvement and to improve early recognition of atypical presentations.

## 2 Case History/Examination

A 4‐year‐old girl of Asian descent presented to Dr. Faisal Masood Teaching Hospital, Sargodha. She had a 1‐week dry cough and 2 weeks of swelling in the face, hands, and feet, which was later attributed to the obesity that is associated with LMBBS. Her vitals at the time of admission were: Pulse: 125 bpm; Temperature: 100.4 F; Respiratory rate: 40/m; SpO_2_: 82% at room air. Her history revealed that she started to gain weight at 6 years of age, and now she is 40 kg at the 95th percentile, height is 110 cm at the 85th percentile, and BMI is 33.1 kg/m^2^. She was developmentally delayed with neck holding at 2 months, sitting with support at 12 months, sitting without support at 18 months, and walking with support at 20 months. An intellectual disability was noticed, and she is not able to speak or understand words properly.

Her vision started to deteriorate at 6 months of age, and now she is not able to see anything properly. She also has a history of polyuria and flank pain since the beginning. There was a consanguineous marriage between the patient's parents; she was delivered through spontaneous vaginal delivery at full term with no NICU admissions. Family history revealed obesity in one parent. The patient has one normal brother.

**FIGURE 1:**
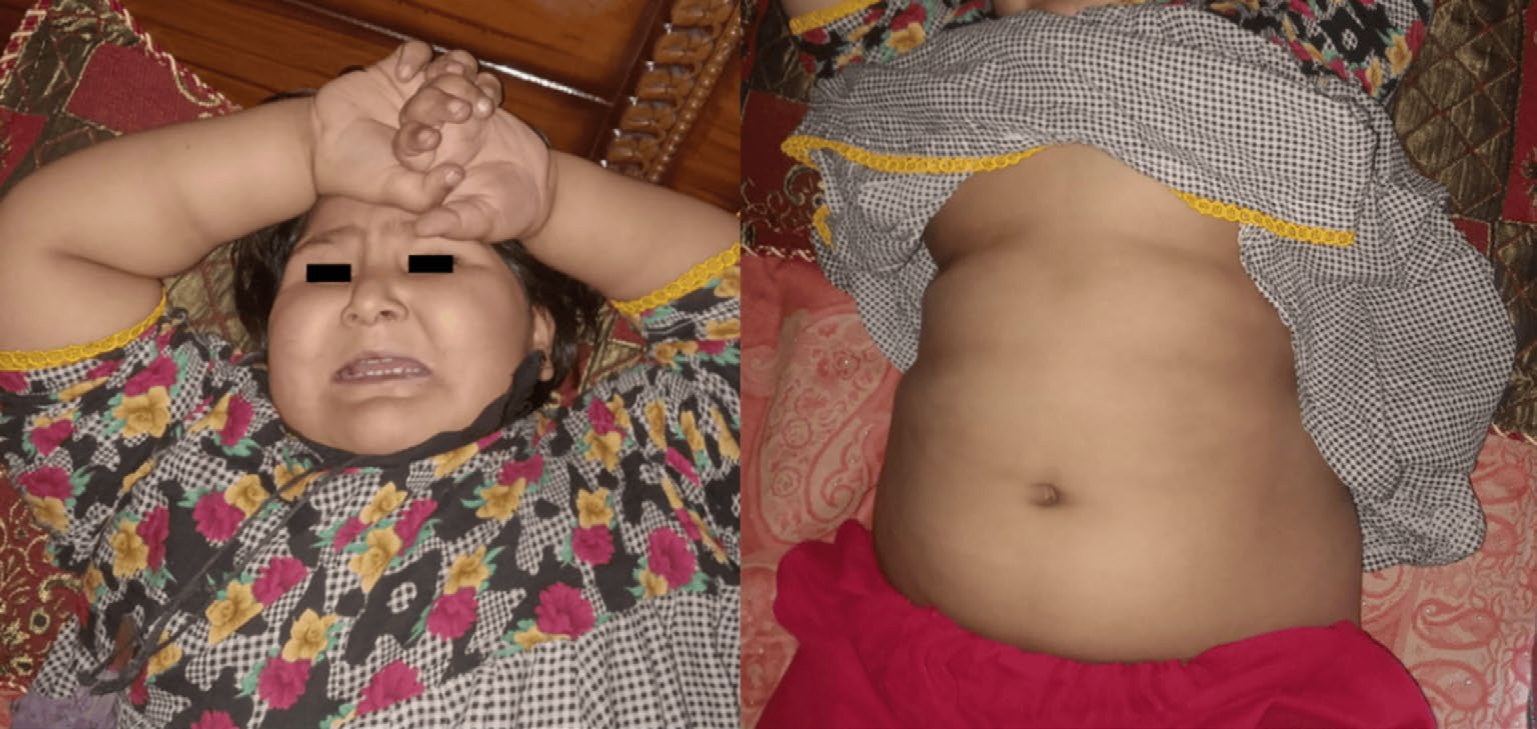
Abdominal obesity and moon facies.

**FIGURE 2:**
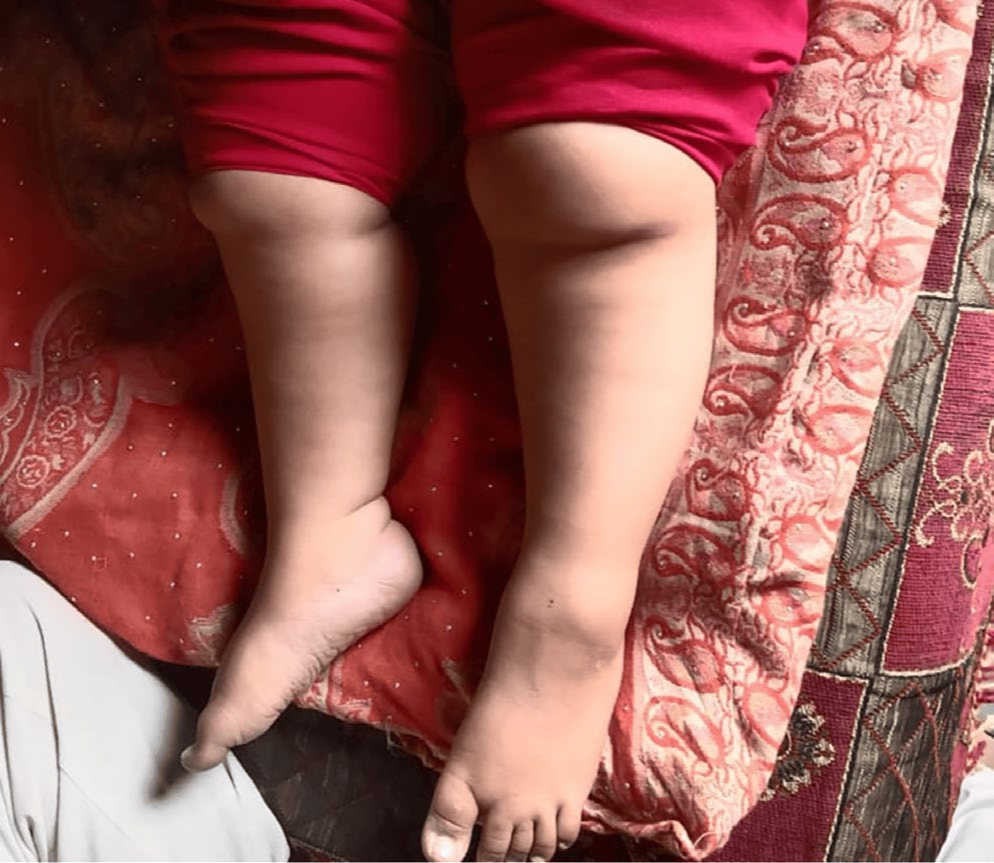
Bilateral non‐pitting edema.

Physical examination revealed obesity and moon facies (Figure 1) with marked edema on the face, upper limbs, and lower limbs, which was bilateral and nonpitting (Figure 2). No polydactyly was present. Oral thrush was present. On abdominal examination, the abdomen was protuberant, with marked ascites (Figure 1). There was mild hepatomegaly. Genitalia were of normal morphology for a female. Signs of respiratory distress were present. There was no pallor or cyanosis. The rest of the examination is unremarkable.

## 3 Methods (Differential Diagnosis, Investigations, and Treatment)

The differential diagnosis for BBS primarily includes other ciliopathies like Alström syndrome, as well as other genetic obesity syndromes like Prader–Willi syndrome, with careful clinical evaluation and genetic testing being crucial for accurate diagnosis; other conditions to consider include Joubert syndrome, McKusick–Kaufman syndrome, Senior–Løken syndrome, and Usher syndrome depending on the presenting features [11].

Lab workup was done to evaluate and rule out other differentials. The following are the abnormal findings: CRP+ve, Urea 59 mg/dL (normal: 18–45 mg/dL), Creatinine 0.8 mg/dL (normal: up to 1.2). Urea and creatinine were measured to evaluate the kidney involvement associated with the syndrome. On ultrasound examination, the liver was enlarged, 13 cm, with normal texture and regular margins. The portal vein and common bile duct are not dilated, and hence, the hepatic causes of the abdominal ascites were ruled out. An echocardiogram was performed due to persistent respiratory distress, which revealed severe tricuspid regurgitation, mild mitral regurgitation, moderate pulmonary regurgitation, with a dilated right atrium and right ventricle, mild pericardial effusion, and marked biventricular apical hypertrophy. These findings were consistent with pulmonary hypertension, supporting it as the cause of the patient's primary complaint. The absence of significant left‐sided dysfunction helped rule out congestive heart failure as a primary cause. Ejection fraction could not be measured accurately due to the high pulse rate of 125 bpm.

To rule out any metabolic abnormalities, an HbA1c was performed, which revealed an HbA1c of 5.7%, falling in the pre‐diabetic range. On ophthalmic examination, fundoscopy revealed pigmentary degeneration with early macular atrophy and vascular attenuation. This helps us rule out Prader‐Willi Syndrome from the differentials.

The patient was managed on furosemide 4 mg IV BD and a combination of spironolactone 40 mg and furosemide 50 mg per oral for edema, hydrocortisone managed with furosemide 100 mg IV, cefuroxime 2 g IV BD, and nebulized using 1 ampule of ipratropium bromide with 2 cc normal saline every 8 h. Later, the patient was also given an injection of levofloxacin 400 mg IV BD.

## 4 Conclusions and Results (Outcome and Follow‐Up)

Even with intense medical interventions, including diuretics, antibiotics, corticosteroids, and mechanical ventilation, the patient's condition worsened. Severe pulmonary hypertension was present at the time of diagnosis, in addition to multi‐organ involvement and concurrent LMBBS, which collectively became overwhelming. Regrettably, though, the patient had an acutely worsening cardiac decompensation, probably due to right heart failure and worsening pulmonary hypertension, and subsequently died.

## 5 Discussion

Laurence–Moon Syndrome (LMS) and BBS are seen as two separate syndromes, and confusion exists between them due to overlapping symptoms, and rigorous genetic testing is required to separate the two [12]. Consanguineous marriage is a possible cause. Its cases in Pakistan are sporadic due to a lack of knowledge, and the majority of the cases go undiagnosed [9].

There is very scarce data available regarding LMBBS. This case is unique in the sense that the patient has pulmonary edema and pulmonary hypertension along with the typical symptoms of LMBBS. The patient presented with atypical findings of mitral regurgitation and pulmonary hypertension. Pulmonary hypertension is a well‐recognized yet seldom reported complication of this syndrome. The pathophysiology involves endothelial dysfunction, vascular remodeling, and inflammation. Comparison with similarly reported cases suggests that presentation can vary. In some cases, it may present as chest pain, dyspnea, and tachypnea, and in some cases, it may also be asymptomatic. Most of the reported children with this diagnosis had idiopathic hypertension with no known cause [4]. Pulmonary hypertension as a secondary symptom of LMBBS, with the finding of increased respiratory rate and tachypnea and coarse crepitations on auscultation, has also been reported [13]. Our patient had tachypnea and increased respiratory rate, pointing towards the suspicion of pulmonary hypertension, which is not a very common presenting feature of the syndrome. Two cases with primary hypertension as the presenting symptom were reported in the literature [4].

It is characterized by primary and secondary clinical features [14]. Primary characteristics include retinal detachment, obesity, polydactyly, genital abnormalities, renal dysfunction, and intellectual disability. Secondary characteristics include ataxia, developmental delay, syndactyly and brachydactyly, speech delay, anosmia, dental abnormalities, congenital heart disease, and diabetes mellitus [14].

Children with average birth weight often develop obesity in early infancy or childhood [4]. Obesity is prevalent in 72% to 86% of the cases [4, 5, 13]. Our patient was of average weight at birth, as self‐reported by the mother, and on presentation, the patient was 40 kg at the age of 4 years.

Renal abnormalities are another diagnostic criterion that is now presenting in increasing frequency [15] in cases involving a range of renal anomalies, including reduced renal parenchyma, small size, calyceal clubbing and blunting, calyceal cysts, medullary cysts, and chronic renal disease [2, 4, 15]. Our patient has an elevated urea and normal creatinine, not indicating any renal involvement.

Mutations in 14 genes are responsible for this autosomal recessive disorder [10]. These genes encode proteins that are required for the proper functionality, maintenance, and regulation of cilia. Mutated genes cause defects in cilia, which then interrupt the signaling pathways. Scientists consider these impaired cilia to be the main cause of LMBBS [10]. A mutation in the BBS1 gene is noted in 25% of the cases, while a mutation in the BBS10 gene is noticed in 20% of cases. Other genes are less responsible [10].

The parents of the patients have a consanguineous marriage with clinical manifestations of obesity, vision deterioration, intellectual disability, and renal dysfunction. Signs of respiratory distress were present. No polydactyly was present. The patient has one normal brother. Lab workup shows CRP+, Urea 59 mg/dL. Ultrasound abdomen shows a liver span of 13 cm. Fundoscopy shows pigmentary degeneration with early macular atrophy. Echocardiography shows mitral regurgitation, pulmonary regurgitation, and tricuspid regurgitation. Her echocardiography also revealed severe pulmonary hypertension secondary to LMBBS, which has not been reported or described before in the literature.

Management of patients with this syndrome involves managing the manifestations of this illness along with early diagnosis and a multidisciplinary approach. Physical therapy, along with exercise aimed at improving muscular strength and reducing the symptoms of spasticity, has proved to be beneficial in the management of the disease. Early diagnosis by genetic analysis is required for effective treatment. Treatment options at later stages are conventional management. Spectacles and visual aids improve visual quality. Physical exercise, dietary modifications, and pharmacological interventions can reduce obesity. For renal dysfunction, dialysis and renal transplantation are advised. Polydactyly is treated surgically. Other symptomatic treatment, regular follow‐ups, and repeated counseling sessions are required for better patient care.

Treatment approaches for pulmonary hypertension in this syndrome are based on expert opinion, and a large‐scale study is required to establish a treatment regimen. However, Phosphodiesterase 5 inhibitors and prostacyclin analogs are beneficial in alleviating the symptoms. The response to treatment can be variable, and a patient might experience some side effects.

## Author Contributions

**Areeba Mariam Mehmood:** conceptualization, data curation, investigation, resources, writing – original draft. **S. M. Salman Hassan:** conceptualization, data curation, investigation, writing – original draft. **Ayesha Malik:** conceptualization, data curation, investigation, writing – original draft. **Muhammad Faizan:** conceptualization, data curation, investigation, writing – original draft. **Muhammad Usama bin Shabbir:** conceptualization, data curation, investigation, writing – original draft. **Aqsa Jabeen:** conceptualization, data curation, investigation, writing – original draft. **Allahdad Khan:** project administration, supervision, visualization, writing – review and editing. **Aseel Kamal:** conceptualization, methodology, writing – review and editing.

## Consent

Written consent from the patient was taken.

## Conflicts of Interest

The authors declare no conflicts of interest.

## Data Availability

Data available on request from the authors.
